# Periodic fluctuation of reference evapotranspiration during the past five decades: Does Evaporation Paradox really exist in China?

**DOI:** 10.1038/srep39503

**Published:** 2016-12-19

**Authors:** Wanqiu Xing, Weiguang Wang, Quanxi Shao, Zhongbo Yu, Tao Yang, Jianyu Fu

**Affiliations:** 1 State Key Laboratory of Hydrology-Water Resources and Hydraulic Engineering, Hohai University, Nanjing 210098, China; 2 College of Water Resources and Hydrology, Hohai University, Nanjing 210098, China; 3 CSIRO Data 61, Private Bag 5, Wembley, WA 6913, Australia

## Abstract

Evidence that the pan evaporation or reference evapotranspiration (ET_0_) as the indicator of atmospheric evaporation capability have decreased along with the continuous increase in temperature over the past decades (coined as “evaporation paradox”) has been reported worldwide. Here, we provide a nationwide investigation of spatiotemporal change of ET_0_ using meteorological data from 602 stations with the updated data (1961–2011). In addition, we explore the trigger mechanism by quantitative assessment on the contribution of climatic factors to ET_0_ change based on a differential equation method. In despite of different shift points regionally, our results suggest that the ET_0_ generally present decadal variations rather than monotonic response to climate change reported in previous studies. The significant decrease in net radiation dominate the decrease in ET_0_ before early 1990s in southern regions, while observed near-surface wind speed is the primary contributor to the variations of ET_0_ for the rest regions during the same periods. The enhancements of atmospheric evaporation capability after early 1990s are driven primarily by recent relative humidity limitation in China. From a continental scale view, as highly correlating with to Pacific Decadal Oscillation, the shift behaviors of ET_0_ is likely an episodic phenomenon of the ocean-atmosphere interaction in earth.

As the only connecting term between water and energy balance, evapotranspiration is the best indicator for the response behavior of transport of water vapor and latent heat as well as hydrological course and the terrestrial ecosystems associated with climate change^1^,^2^. Along with significantly increasing near-surface air temperatures, decreasing pan evaporation (ET_pan_), potential evapotranspiration (ET_p_) and reference evapotranspiration (ET_0_) have been reported in many regions worldwide and continuously documented by many studies since the first publication by Peterson *et al*. in 1995^3^. This phenomenon has been denoted as the “evaporation paradox”^4^. However, the interpretations for this phenomenon by different scholars are various in different regions, mainly including the following: (1) decreasing ET_p_ or ET_0_ may actually be a strong indication of increasing terrestrial evaporation due to the complementary relationship between the actual evaporation and potential evaporation^5^,^6^,^7^,^8^,^9^; (2) positive effect on evapotranspiration of increasing temperature could be offset by widespread “*dimming*” and “*stilling*” phenomena due to the fact that evaporative process is also primarily driven by radiative and aerodynamic components^4^,^10^,^11^.

Decreases in ET_p_, ET_pan_, and ET_0_ have also been reported to be occurring simultaneously in China or some regions of China with increasing trends of air temperature during the past decades^12^,^13^,^14^,^15^,^16^,^17^,^18^,^19^. Reduced wind speed and shortened sunshine duration are generally considered to be responsible for these phenomena^9^,^20^,^21^,^22^. However, some of conclusions drawn from previous studies on evapotranspiration trends are based on the time series data ended around 2000. Whether the deceasing trends in potential evaporation are still true when looking at the longer record with updated data should also be addressed. In addition, most studies mainly focus on the general trends without the consideration of the abrupt change in the temporal processes. Overall trend estimations may leave out detailed information and thus make these revealed changing patterns deviate from the real situation. Recently, contrary to the famous “evaporation paradox”, an increase in ET_pan_ in China along with continuously rising temperature during the recent two decades was identified by Liu *et al*.^23^ with more recent observations. This phenomenon was further regionally verified in the hyper-arid region of Northwest of China by Li *et al*.^24^. Moreover, Wang *et al*.^25^ suggested that there was actually a zigzag increasing-decreasing-increasing pattern of regional average ET_0_ series with two joint points in 1973 and 1993 in the Tibetan Plateau. It is therefore worthwhile to re-examine the ET_0_ patterns in larger area with different climatic and geomorphologic conditions based on the updated data. However, nationwide investigation on the changing patterns of ET_0_ and its related trigger mechanism is not available.

The goal of this study is to examine whether the “evaporation paradox” is still true by investigating the response of ET_0_ to changing climatic environment based on recently updated nationwide observation in China. Specifically, we begin by identifying the homogenously sensitive regions of ET_0_ using a rotated empirical orthogonal function clustering method. Then we assess the spatial patterns of ET_0_ trends and analyze the abrupt change in average series for individual homogenous regions. Meanwhile, special efforts are further made in characterizing the underlying aerodynamic and radiative driving mechanism for the ET_0_ changes. Finally, the driving mechanisms under natural large atmosphere system are explored by identifying the correlation between changes in ET_0_ and Pacific Decadal Oscillation.

## Results

### Identification of homogenous regions of ET_0_

**Figure 1:**
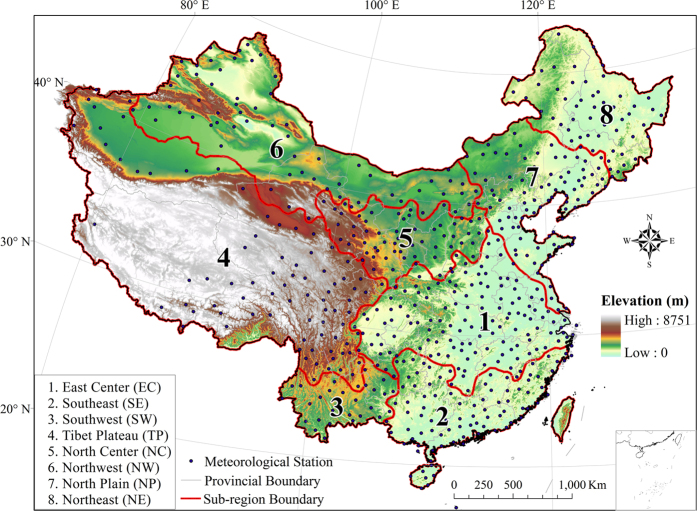
The sub-regions identified by REOF analysis and the spatial distribution of 602 meteorological stations in Mainland China. This figure was created using the ArcGIS 9.3 (http://www.esri.com/software/arcgis/arcgis-for-desktop).

To intensively characterize the spatial anomalies of ET_0_ change nationwide, it is necessary to make a reasonable partition to the whole country. Traditionally, the whole China is always divided into eight climatic regions according to the latitude and longitude, and the climate regions are roughly coincide with the socioeconomic division^13^,^23^. However, there may be some uncertainty in sub-region selection based on visual inspection of the geographical distribution or administrative practices due to its vulnerability to subjective discretion^9^. This is especially true for the study of ET_0_ changing patterns due to the fact that ET_0_ involve in multifarious factors including geomorphologic features, topography factors and climate conditions. Therefore, the rotated empirical orthogonal function (REOF) method, a widely used clustering algorithm to objectively capture realistic spatial information, is employed in the current study to identify the homogenous regions regarding ET_0_ in the whole China. The first 11 EOFs together explain 80.8% of the variance and the first 8 EOFs together can explain 73.7% of the variance. The fact that more than 70% of the total variability is captured in the first 8 EOFs indicates that the complexity of spatial patterns of ET_0_ all over the country can mostly be explained by a small number of spatial structures. The percentages of variance of the new set of REOF modes generated by rotating the first eight loading vectors of the initial EOFs (Table S1) and correspondent isolines of the loading factor values (Figure S1) suggest that Mainland China can be categorized into eight homogenous regions (Fig. 1) by REOF analysis based on the annual ET_0_ series from 602 stations for 1961–2011. Eight leading modes deduced sub-regions can roughly be described respectively as following: East Center China (EC), Southeast China (SE), Southwest China (SW), Tibet Plateau (TP), North Center China (NC), Northwest China (NW), North China Plain (NP), and Northeast China (NE). In term of the regional average series of these eight homogenous regions, investigation on temporal patterns and their attributions across the whole China are presented in following sections.

### Multidecadal changing patterns in ET_0_

**Figure 2:**
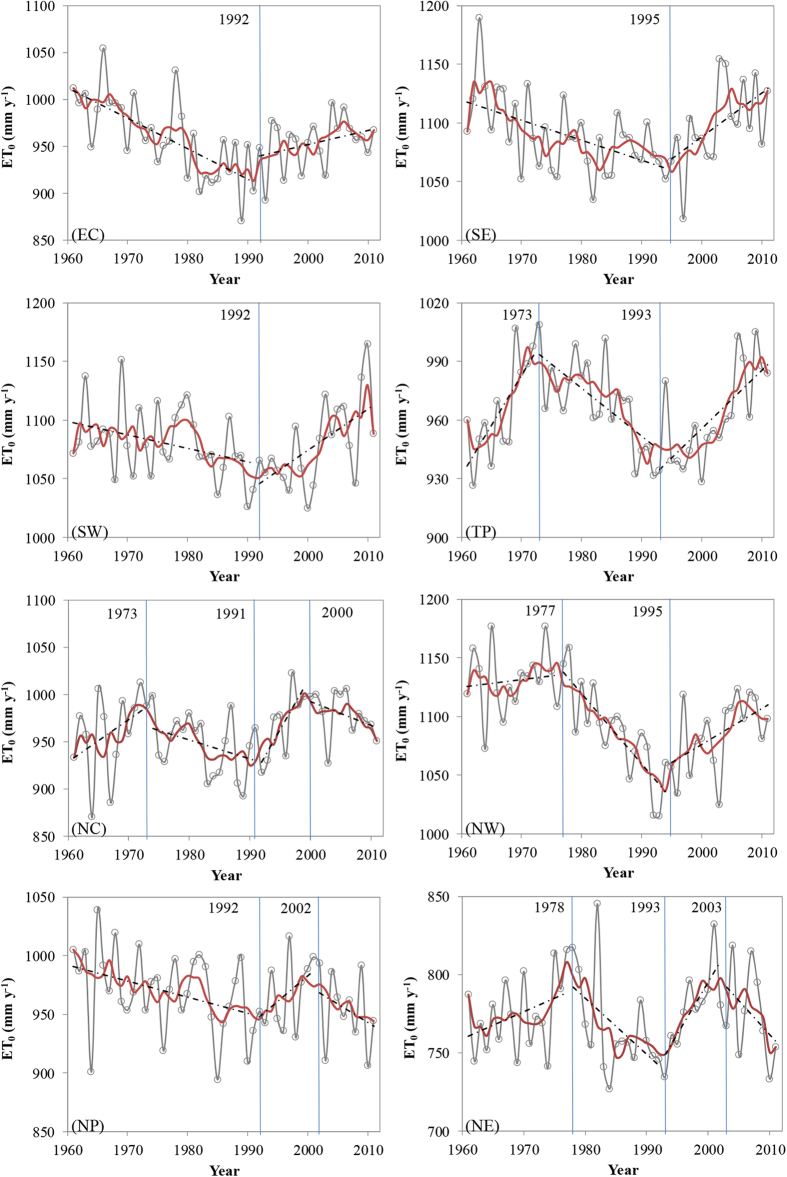
Long term variations and abrupt change of annual reference evapotranspiration (ET_0_) for the eight subregions during 1961–2011. The grey solid line with circles and the red line denotes the observed series and the smoothed series, respectively. The blue vertical line give the location of change points and the black dash lines are the segmented linear trends fitted to corresponding periods.

**Figure 3:**
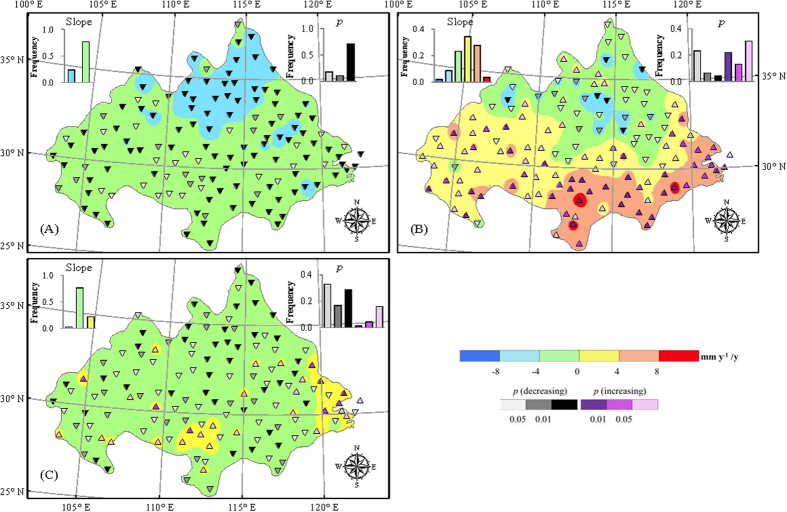
Spatial distributions of the change rate of reference evapotranspiration (ET_0_) and significance levels of the change in the first sub-region during the three periods ((**A)**: 1961–1991; (**B)**: 1992–2011; and (**C**): 1961–2011). *p*(decreasing) and *p*(increasing) are the *p* values of the decrease and increase in the annual reference evapotranspiration (ET_0_), respectively, which are divided into three levels: *p* < 0.01, 0.01 <*p* < 0.05, and *p* > 0.05. The insets show the frequency distributions of change trends of reference evapotranspiration (ET_0_) (left) and different significance levels (right). This figure was created using the ArcGIS 9.3 (http://www.esri.com/software/arcgis/arcgis-for-desktop).

We conduct trends and change point analysis for the area-averaged ET_0_ in the resulted eight sub-regions by the means of the segmented regression model (shown in Fig. 2 and Table 1). The overall impression from the temporal patterns in ET_0_ series is that shift trends can be evidently found in all eight sub-regions, although the turning years and the number of change points are regionally different. The decreasing-increasing (DI) patterns (with one joint point) are detected in EC, SE and SW regions with joint points happening at about 1992, 1992 and 1995, respectively. The zigzag increasing-decreasing-increasing (IDI) patterns (with two joint points) are depicted in TP (1973 and 1993) and NW (1977 and 1995) regions. With three joint points, approximating to periodic variation, more complex zigzag increasing-decreasing-increasing-decreasing (IDID) patterns are identified in NC (1973, 1991 and 2000) and NE (1978, 1993 and 2003) regions. It should be noted that although only two points of 1992 and 2002 are found in NP region, the overall changing pattern of decreasing-increasing-decreasing for ET_0_ are similar to NC and NE regions, especially for the recent two decades. Interestingly, irrespective of complex shift trends reflected by different number of joint points and turning modes, multidecadal changing features in ET_0_ are characterized by an evident spatial clustering partition. This clustering partition can roughly indicates that DI patterns mainly occur in southern region in China with a humid climate, whereas IDI mostly emerge in western Plateau regions with a cold and drought climate and IDID mainly appear in north and northeast regions with a cold climate. We also investigate the trends for the 602 stations during the segmented periods according to the corresponding regional change points. Taking EC region for example, as shown in Fig. 3, annual ET_0_ at all stations decrease during 1961–1991 by 0–8 mm yr^−1^ (over 23% stations by 4–8 mm yr^−1^), of which over 80% stations are statistically significant at 95% confidence level (*p* < 0.05). As for as the second segmented period, from 1992–2011, more than 65% stations (over 30% stations by 4–12 mm yr^−1^) are dominated by increasing trends in ET_0_ despite that only 35% stations are statistically significant at 95% confidence level (*p* < 0.05). Such extremely distinct spatial patterns in ET_0_ during the adjacent segmented period are also identified in other homogeneous regions (shown in Figure S2–S8), giving us confidence in detecting multidecadal changes in ET_0_ with shift trends in China.

### Driving factors of changing ET_0_

**Figure 4:**
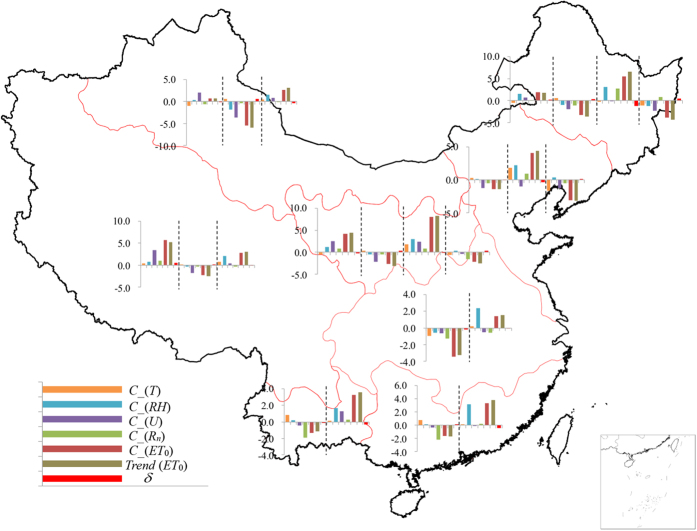
The contribution of annual climatic variables change to reference evapotranspiration (ET_0_) change in each sub-region. This figure was created using the ArcGIS 9.3 (http://www.esri.com/software/arcgis/arcgis-for-desktop).

The “evaporation paradox” is stated based on measured pan evaporation^3^, therefore, the correlation analyses of ET_0_ with ET_pan_ (data collected from183 stations with the same records of ET_0_) is conducted before the contribution assessment of climatic factors. The close relationship between annual ET_pan_ and ET_0_ with correlation coefficient (*R*) of more than 0.9 suggests that ET_pan_ is a good indicator of ET_0_ across the whole China (Figure S9). We identify the driving factors of changing ET_0_ by quantifying the contribution of climatic factors to ET_0_ change using partial derivatives (see Equation 10). For the eight homogeneous regions, the maximum relative error *ρ*(*δ*) representing the ratio of error *δ* to observed trend is found in NC region during the fourth segmented period of 2000–2011 with the value of −14.78%. The minimum *ρ*(*δ*) is only 1.70%, which occurs in NP region during the first segmented period of 1961–1991 (Table S2). The calculated ET_0_ trends for all regions during all the segmented periods match very well with those detected from the computed ET_0_ with P-M method (always regarded as observed ones) with quite satisfactory *R*^2^ value of 0.99. The high accordance of calculated ET_0_ trends with estimated ones reflected by high *R*^2^ and low error indicates the differential equation method is applicable to quantify the contributions of climatic variables to changes in ET_0_. The contributions of climatic factors to the trends in ET_0_ in categorized eight sub-regions during different periods are shown in Fig. 4. Taking NC regions with most complex shift trends in ET_0_ as an example, during 1961–1972, the decreasing air temperature (T) causes a decrease of ET_0_ at the rate of 0.42 mm yr^−1^. Meanwhile, the increase of wind speed (U) and net radiation (R_n_) as well as the decease of relative humidity (RH) lead to the increase of ET_0_ at the rate of 2.53 mm yr^−1^, 0.85 mm yr^−1^ and 1.18 mm yr^−1^, respectively. Consequently, U is the dominant factor for the change in ET_0_ in NC region during 1961–1972. Likewise, U, RH and R_n_ are responsible for the decrease of ET_0_ during 1973–1990, the increase of ET_0_ during 1991–1999 and the decrease of ET_0_ during 2000–2011 in NC region, respectively. Overall, although the change in ET_0_ of the whole country is characterized by complicated spatial and temporal variability in the dominating contribution factors, some interesting phenomena can be summarized as below. Before the early 1990s, the decreases of ET_0_ in EC, SE and SW regions, mostly located in South China, are mainly attributed to the decrease of R_n_. While for the rest regions including TP, NC, NW, NP and NE, mainly distributed in western and northern part of China, the changes of ET_0_ before the early 1990s (all consist of increasing period before 1970s and subsequent decreasing period except NP region) mainly resulted from the changes of U, except the NE region during 1961–1977 with the dominating factor RH. From early 1990s to 2011, the ET_0_ in EC, SE, SW, TP and NW regions present monotonous increase. While for the NC, NP and NE regions, changes in ET_0_ are separated into two stages, i.e., increases from early 1990s to around 2000 and decreases after 2000. Although R_n_, T and U play respectively the most import role in the decrease of ET_0_ at NC, NP and NE regions after around 2000, decreasing trends in RH is the most crucial factor for the increasing trends in ET_0_ detected after the early 1990s in all the eight sub-regions.

### The linkage between multidecadal patterns of ET_0_ and the Pacific Decadal Oscillation (PDO)

**Figure 5:**
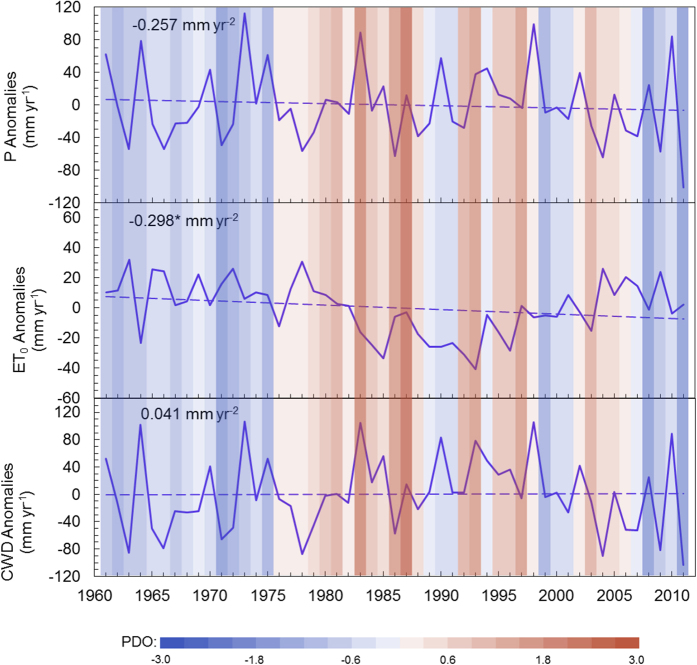
Yearly anomalies of P, reference evapotranspiration (ET_0_) and CWD (P- ET_0_) from 1961 to 2011; the linear trends of annual values of the three variables are calculated by the simple linear regression and shown as dashed lines, *p < 0.1, **p < 0.05. The Pacific Decadal Oscillation index, PDO, is shown with vertical color shading, where red and blue shades denote respective positive and negative phases.

In order to investigate the decadal characteristic of ET_0_ in China and seek for the possible driving mechanisms, the regional mean anomalies of annual ET_0_, precipitation (P) and the climatic water availability (CWD, defined as P minus ET_0_) as well as its relationship to the Pacific Decadal Oscillation index (PDO) are analyzed. Although as a whole, the regional ET_0_ record shows a significant decrease trend of 0.298 mm yr^−2^ (*p* < 0.1) during the past 50-years period, the evolving process of continental mean ET_0_ can be clearly divided into three stages. From 1961 to 1979, ET_0_ present a slight but not significant decline (0.008 mm yr^−2^) with a high mean value. A prolonged significant decline starts in the year of 1980 and ends in the year of 1995, being contrary to the previously reported upward actual evapotranspiration in this period^26^,^27^,^28^. This may partly be explained by the complementary relationship^29^ between ET_0_ and actual evapotranspiration. After 1996, a pronounced recovery growth rate in ET_0_ can be clearly revealed. An overall oscillation in regional mean ET_0_ is found in China, which was not revealed by the previous studies, suggesting that the variation of ET_0_ from 1961 to 2011 should be a phenomenon of earth’s atmospheric circulation. PDO is considered as the important earth’s climatic driving power for the continental hydro-meteorology abnormality such as shift of dry/wet in Asia, especially in China^30^,^31^,^32^,^33^. The PDO driven climate oscillations impart clear influences to the regional ET_0_ in China as a whole, indicated by significant negative correlation between annual PDO index and annual mean ET_0_ (*r* = −0.384; *p* < 0.001). Years with high ET_0_ mostly coincide with PDO cold phase although ET_0_ responses to PDO may differ in different sub-regions in China. On the other hand, years with low ET_0_ correspond to PDO warm phase conditions (Fig. 5). The regional mean P in China present large temporal variability with a negative but not significant trend of 0.257 mm yr^−2^ (*p* = 0.564) during 1961–2011, while the CWD present a significantly no trend although the slope is slightly positive with the value of 0.041 mm yr^−2^ (*p* = 0.938), suggesting that there is a weak tendency to reduce the deficit between atmospheric moisture requirement and available water supply for evapotranspiration. Despite of the strong relationship between regional ET_0_ and PDO activities, the CWD is not significantly correlated with PDO (*r* = 0.164, *p* > 0.1) due to the weak relationship between P and PDO (*r* = 0.045, *p* > 0.1).

## Discussion

The “evaporation paradox”, reported in many regions of the world including China^15^, has drawn a considerable attention to explore the reason of the decreases in ET_pan_ and/or ET_0_ with increases in air temperature. However, with more recent observations or longer record, an increase in ET_pan_ and/or ET_0_ since 1980s or 1990s has been found in China or some regions of China^23^,^24^,^34^,^35^,^36^. Specially, a zigzag increasing-decreasing-increasing pattern with two joint points in 1973 and 1993 has been identified in our previous study on the changes in ET_0_ across the Tibetan Plateau^25^. However, the results from most previous studies on ET_0_ change and driving mechanism are only based on subjective sub-regions selection. Meanwhile, the natural large atmosphere system triggering mechanisms for ET_0_ change have not been investigated in previous studies. Consequently, a nationwide investigation in China is naturally performed in this study to detect the real multidecadal changing patterns of ET_0_ and the correlation with PDO with extended data from 1961 to 2011. The temporal patterns of ET_0_ in eight homogenous regions identified by REOF evidently reveal that there is no monotonous decreasing trend in ET_0_ along with the continuously increasing temperature for the entire period (from 1961 to 2011), suggesting “paradox” phenomenon just exist in a certain stage of the multidecadal changes in ET_0_ in China. With one turning point, the finding that decreasing trends of ET_0_ reverse into increasing trends in early 1990s across EC, SE and SW regions, is in agreement with region studies on ET_pan_/ET_0_ by Liu and Zhang (2012)^37^ and Li *et al*.^24^. When other sub-regions are considered, coarse periodic behaviors of ET_0_ change with two/or three turning point seems to be reasonable as compared with total decreasing-increasing patterns of evaporation in entire China reported by Cong *et al*.^38^ and Liu *et al*.^23^. The combined effects of climatic variables to ET_0_ changes are revealed in all the sub-regions with the sensitive analysis, and are effective with a good agreement between the observed and calculated ET_0_ trends (see Table S2). Although relative humidity is the most sensitive variable (followed by net radiation, wind speed and air temperature) for all the period as well as all the sub-regions except SW and TP region, there are different dominating factors for ET_0_ change because the contributions of climate factors depend on the combined effect between sensitivity and trends of a variable itself^9^. Decline of net radiation only plays the important role for the ET_0_ decrease before early 1990s rather than the entire term in EC, SE and SW regions located in south China, highlighting the potential implications of “global dimming” in China over a roughly 1960–1990 period and a recovery (coined with “global brightening”) thereafter^39^,^40^, which is consistent with previous studies in China^13^,^20^,^23^,^38^,^41^, the United States^3^, the northeast of India^42^, and Greece^43^. There is consensus among many researchers that cloud coverage and aerosol, which are not completely independent variables with interaction in various ways^44^, are considered as the most likely candidates for the explanation of global dimming and brightening^39^,^45^,^46^,^47^,^48^. Particularly strong evidence for aerosol effects on surface solar radiation with increasing air pollution in China were noted in various studies^49^,^50^,^51^,^52^. As far as the other regions with larger inter-annual variability in ET_0_ are concerned, variation of wind speed generally plays more decisive role in the change of ET_0_ from 1961 to early 1990s, in line with the evidences reported in many places and summarized by McVicar *et al*.^11^. Although the dynamic mechanism of recent slow-down in near-surface global winds is complicated, it can generally be attributed to the increases of terrestrial surface roughness^53^,^54^ or large scale atmospheric circulations^55^,^56^,^57^,^58^. Focusing on the periods after 1990s, our preliminary analyses have shown that dominating factors for the increases of ET_0_ transited from net radiation and wind speed to relative humidity for all the sub-regions. This coincides with the recent regional study in the arid regions of northwest china by Li *et al*.^24^ and national wide study in China by Cong *et al*.^38^. A global evidence of reduction in surface atmospheric humidity revealed in this study in national scale was presented by Simmons *et al*.^59^. They inferred the recent reduction in relative humidity over land may be due to limited moisture supply from the oceans. For the purpose of simplification, the systemic errors of contribution analysis reflected by the differences between observed ET_0_ and complied trends may be due to the interactions among climatic factors (which were not considered here but may exist in practice) and ignorance of other factors such as aerosol and dust.

From global perspective, there is an indication for a shift that occurred in the late 1970s with minor change occurred around 1990 and 2000 for PDO^60^,^61^. Having the strong correlation with PDO, the decadal variations of ET_0_ in China with two turning points during 1961–2011 are likely dominated by the episodic dynamics of climatic system, indicating that the recent ET_0_ trends in China reflect PDO conditions and are not the consequence of a persistent reorganization of the hydro-meteorological cycle. The similar conclusions have also been obtained from the global analysis for continental evaporation^27^,^28^. Consequently, the widely reported “evaporation paradox”, a counterintuitive behavior, representing that evapotranspiration decrease despite the increase of air temperature, seem to be more reasonably explained as part of a climate oscillation, at least in China^33^. The emerging picture of enhanced of ET_0_ in southern China highlights the possible threat posed by acceleration of the terrestrial hydrological cycle to water resources management and food security in an enhanced-greenhouse-affected climate.

## Materials and Methods

### Data

Daily measurements of air temperatures (minimum, maximum and average) at 2 m height, relative humidity, wind speed at 10 m height, and sunshine duration obtained from the National Climatic Centre (NCC) for 602 ground-based stations in China during the period 1961–2011, provided by the National Meteorological Information Centre of China (NMIC) of the China Meteorological Administration (CMA), are used for estimating ET_0_ by Penman-Montieth method. Wherein, wind speed was adjusted to 2 m height by virtue of wind profile relationship proposed by Allen *et al*.^62^. Net radiation at 51 stations was available. Moreover, precipitation data from these meteorological stations were also collected.

### Food and Agriculture Organization (FAO) Penman-Montieth (P-M) Method

From the reference surface defined as “a hypothetical reference grass with an assumed crop height of 0.12 m, a fixed surface resistance of 70s m^−1^ and an albedo of 0.23”, the daily reference evapotranspiration (ET_0_) is estimated by the Penman-Montieth (P-M) combination method, which is recommended by the FAO as a standard to calculate ET_0_ wherever the required input data are available and is proven to have good performance for various climatic conditions worldwide^9^. The P-M equation can be expressed as (Allen *et al*.)^62^:


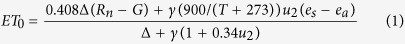


where *ET*_0_ is the reference evapotranspiration (mmd^−1^), *R*_*n*_ is the net radiation at the crop surface (MJ m^−2^ d^−1^), *G* is the soil heat flux density (MJ m^−2^ d^−1^), *T* is the mean daily air temperature (°C), *u*_2_ is the daily average wind speed at 2 m above ground level (ms^−1^), *e*_*s*_ is the saturation vapor pressure (kPa), *e*_*a*_ is the actual vapor pressure (kPa), *e*_*s*_ − *e*_*a*_ is the saturation vapor pressure deficit (kPa), Δ is the slope of the saturated vapor pressure in relation to air temperature (kPa °C ^−1^) and γ is the psychrometric constant (kPa °C ^−1^).

As the difference between incoming net shortwave radiation (*R*_*ns*_) and outgoing net longwave radiation (*R*_*nl*_), *R*_*n*_ can be expressed as:


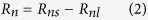


*R*_*ns*_ is derived from the balance between incoming and reflected solar radiation and is given by:


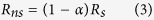


where *a* is the albedo or canopy reflection coefficient, which is 0.23 for the hypothetical grass reference crop.

*R*_*s*_ can be estimated from sunshine duration (or hours of sunshine) with the help of the Angstrom formula


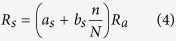


where *R*_*s*_ is the solar or shortwave radiation (MJ m^−2^ d^−1^), *n* is the actual duration of sunshine (h), *N* is the maximum possible duration of sunshine or daylight hours (h), (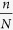 is thus the relative sunshine duration), *R*_*a*_ is the extraterrestrial radiation (MJ m^−2^ d^−1^), *a*_*s*_ and *b*_*s*_ are the Angstrom coefficients. We calibrate the Angstrom coefficient using the observation of *n* and *R*_*s*_ from 51 stations widespread the whole China. Meanwhile, *Ra* and *N* are computed based on date and latitude according to the equations provided by the FAO P-M method. Overall, the high *R*^2^ values (see Table S3) between observed and estimated *R*_*s*_ indicate that the Angstrom model is suitable for daily global radiation estimation in China. For stations with no observation of solar radiation but sunshine duration, a_*s*_ and *b*_*S*_ are estimated by Kringing interpolation method.

*R*_*nl*_ is based on water vapor, clouds, carbon dioxide and dust are absorbers and emitters of longwave radiation, and can be estimated by


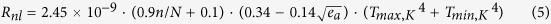


where *T*_*max,K*_ and *T*_*min,K*_ are respectively maximum and minimum absolute temperatures during the 24-hour period, *K *= °C + 273.16.

The soil heat flux, *G*, is the energy that is utilized in heating the soil. *G* is positive when the soil is warming and negative when the soil is cooling. The soil heat flux is small compared to Rn and may often be ignored in daily evapotranspiration estimating.

### Trend analysis and breakpoint identification

A simple linear regression method was used to calculate the slope of linear least squares regression line fit to the inter-annual variation of ET_0_ and other climatic variables inputted in the P-M equation. The significance of changes of these variables across China was mapped and assessed with the help of the two-tailed significance tests. The segmented regression with constraints method developed by Shao and Campbell^63^, which can detect both shift trends and step changes simultaneously without knowing the number of trend segments and change points and their location over time^64^, is employed to investigate the possible turning points of the regional average ET_0_ series across China. The method was summarily described as follow^63^,^64^,^65^:

Assuming that *x*_*i*_ means the observed variable at time *t*_*i*_ (*i* = 1, 2, …, *N) t*_*i*_, the regression can be written as


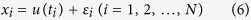


where the errors {*ε*_*i*_} (*i *= 1, 2, …. *N*) are assumed to be independent and identically distributed as *N*(0, *σ*^2^). For *L* abrupt change points *r*_1_ < *r*_2_ < ···*r*_*L*_, resulting in *L* + 1 segments, the model can be written as


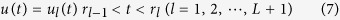


with *r*_0_ = 0, *r*_*L*+1_ = ∞ and *u*_*l*_(*t*) are the deterministic part quantifying the trends. Suppose that there are *j*_*l*_ join points {*J*_*l*., *k*_} (*k* = 1, 2, …. *j*_*l*_) in the *i*th segment between the break points *r*_*l*−1_ and *r*_*l*_, *u*_*l*_(*t*) can be defined as:


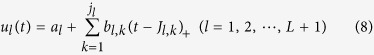


with *J*_*l*, 0_ = *r*_−1_, where (*t*−*J*_*l, k*_)_+_ = *u*_+_ is defined as *u*_+_ = *u* if *u* ≥ 0. A modified Akaike’s information criterion (AIC*c*), derived by Hurvich and Tasi^66^ with *c* standing for the second order correction^67^, is employed to select an optimal numbers of break and join points and their locations, and the optimal model can be obtained by minimizing AIC*c*.

### The Rotated Empirical Orthogonal Function (REOF) method

To characterize in detail the spatial variability of ET_0_ at nationwide scale and identify the homogenous regions, we applied REOF to analyze the most dominant spatial patterns. The aim of the EOF method is to find a relatively small number of independent variables conveying as much of the original information as possible without redundancy by decomposing a multivariate data set into uncorrelated linear combination of separate function of the original variables. However, the physical interpretability of the obtained patterns is sometimes a matter of controversy because of orthogonality in both space and time^68^. This limitation has brought about the development of the rotated empirical orthogonal function (REOF)^69^, which can cluster within each mode a small number of high valued variables and a large number of near-zero value variables through a rotation to simple structure. In a comparison study by Kim and Wu^70^, REOF was found to be better in dividing climatic patterns. In this paper, with maximizing the variance of the squared correlation between each rotated principal component (RPCs) and each variable, the varimax REOF method was chosen to give the simplest pattern description while explaining the maximum amount of variance.

### The differentiation equation method

For the function *y* = *f*(*x*_1_, *x*_2_, ···), the variation of the dependent variable *y* over the time *t* can be mathematically written by a differential equation as


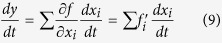


where *x*_*i*_ is the *i*th independent variable and 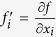


Assuming that *y* and *x*_*i*_ are the time series variables, 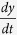 and 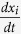 should be the long-term trend for *y* and *x*_*i*_, respectively. The term of 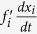, the product of long-term change in *x*_*i*_ and the partial derivative, can then be considered as the contribution of change in *x*_*i*_ to the long-term variation of *y*. Thus, following P-M equation for ET_0_ estimation, contributions of changes in key climatic factors to ET_0_ variation can be approximately as^55^:


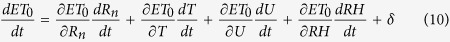


where 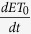 is the long-term change in ET_0_ estimated by the above partial derivatives. 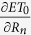

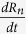
, 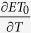

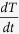
, 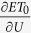

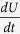
 and 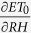

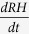
 are respectively the contributions to the long-term change in ET_0_ due to the variation in *Rn, T, U* and *RH. δ* is the error term.

### The sensitive coefficient

For such a multi-variable model of P-M equation, it is difficult to compare the sensitivity by partial derivatives due to the different dimensions and ranges of values for related variables. Hence, the partial derivative is transformed into a non-dimensional form in term of McCuen^71^ and Beven^72^ to express the sensitivity of the climatic variables mathematically:


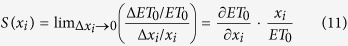


where *S*(*x*_*i*_) is the sensitivity coefficient of ET_0_ related to *x*_*i*_, the *i* th variable. Being first employed by McCuen^71^, the sensitivity coefficient has been widely used in evaluating the climatic response of evapotranspiration, especially during recent two decades^73^,^74^,^75^,^76^,^77^.

## Additional Information

**How to cite this article**: Xing, W. *et al*. Periodic fluctuation of reference evapotranspiration during the past five decades: Does Evaporation Paradox really exist in China? *Sci. Rep.* **6**, 39503; doi: 10.1038/srep39503 (2016).

**Publisher's note:** Springer Nature remains neutral with regard to jurisdictional claims in published maps and institutional affiliations.

## Supplementary Material

Supplementary Information

Supplementary Dataset 1

## Figures and Tables

**Table 1:** Trend slope of reference evapotranspiration (ET_0_) and meteorological variables in linear regression analysis, and the dominating factor result to ET_0_ change by attribution analysis.

Sub-region	Variables	Trend Slope			Sub-region	Variables	Trend Slope			
EC	*Periods*		*1961–1991*	*1992–2011*	NC	*Periods*	*1961–1972*	*1973–1990*	*1991–1999*	*2000–2011*
	*ET*_*0*_		−3.2646**	1.5064		*ET*_*0*_	4.4246	−3.0376**	8.187*	−2.538
	*T*		−0.0048	0.0335**		*T*	−0.0142	0.0233	0.1705**	0.0185
	*RH*		0.0342	−0.2420**		*RH*	−0.1565	0.0474	−0.3429	−0.0562
	*U*		−0.0185**	−0.0113**		*U*	0.0355**	−0.0286**	0.0056	−0.0041
	*R*_n_		−0.0194**	−0.0084		*R*_n_	0.005	−0.0087	0.0087	−0.0225**
	Dominating Factor		*R*_n_	*RH*		Dominating Factor	*U*	*U*	*RH*	*R*_n_
SE	*Periods*		*1961–1994*	*1995–2011*	NW	*Periods*	*1961–1976*	*1977–1994*	*1995–2011*	
	*ET*_*0*_		−1.7178**	3.7568**		*ET*_*0*_	0.673	−5.9695**	3.0873**	
	*T*		0.0069	0.014		*T*	−0.0338	0.0275	0.0234	
	*RH*		−0.006	−0.3195**		*RH*	−0.0336	0.1075*	−0.1129	
	*U*		−0.0113**	−0.0032		*U*	0.0210**	−0.0402**	0.0055**	
	*R*_n_		−0.0162**	0.0026		*R*_n_	−0.001	−0.0044*	−0.0035*	
	Dominating Factor		*R*_n_	*RH*		Dominating Factor	*U*	*U*	*RH*	
SW	*Periods*		*1961–1991*	*1992–2011*	NP	*Periods*		*1961–1991*	*1992–2001*	*2002–2011*
	*ET*_*0*_		−1.1510**	3.5493**		*ET*_*0*_		−1.3780**	4.3713	−3.1608
	*T*		0.0110*	0.0253*		*T*		0.0183*	0.0571	−0.1057*
	*RH*		−0.0298	−0.1921**		*RH*		−0.0118	−0.0939	−0.0467
	*U*		−0.0002	0.0156**		*U*		−0.0177**	−0.0187**	−0.013
	*R*_n_		−0.0131**	0.0027		*R*_n_		−0.0084**	0.0137	−0.0047
	Dominating Factor		*R*_n_	*RH*		Dominating Factor		*U*	*RH*	*T*
TP	*Periods*	*1961–1972*	*1973–1992*	*1993–2011*	NE	*Periods*	*1961–1977*	*1978–1992*	*1993–2002*	*2003–2011*
	*ET*_*0*_	5.1585**	−2.4605**	2.9879**		*ET*_*0*_	1.7502	−3.5712*	6.5942**	−4.3362
	*T*	0.0282	0.0084	0.0525**		*T*	−0.0026	0.0809**	−0.0166	−0.0938
	*RH*	−0.1289**	0.0393	−0.2910**		*RH*	−0.0295	0.0899	−0.1885	0.1567
	*U*	0.0497**	−0.0239**	0.0015		*U*	0.0034	−0.0381**	−0.002	−0.0273**
	*R*_n_	0.0161**	−0.0052*	−0.0052		*R*_n_	0.0046	−0.0089	0.0242*	0.0189
	Dominating Factor	*U*	*U*	*RH*		Dominating Factor	*RH*	*U*	*RH*	*U*

Note: Trend slope of linear regression of *ET*_0_, *T, RH, U*, and *R*_n_ are expressed in mm y^−1^/y, °C/y, %/y, m s^−1^/y, and W m^−2^/y, respectively, and “*”represent significance at *p* = 0.10, “**”represent significance at *p* = 0.05.
