# Supplementary material for: Periodic fluctuation of reference evapotranspiration during the past five decades: Does Evaporation Paradox really exist in China?
Source: Sci Rep. 2016 Dec 19;6:39503. doi: 10.1038/srep39503 (PMC5172234; doi:10.1038/srep39503)
Supplement: Supplementary Information [file srep39503-s1.pdf]

Supplementary Information

Periodic fluctuation of reference evapotranspiration during the past five decades: Does Evaporation Paradox really exist in China?

Wanqiu Xing[a,b], Weiguang Wang[a, b]*, Quanxi Shao[c], Zhongbo Yu[a], Tao Yang[a,b], Jianyu Fu[a,b]

a. *State Key Laboratory of Hydrology-Water Resources and Hydraulic Engineering, Hohai University, Nanjing 210098, China*

b. *College of Water Resources and Hydrology, Hohai University, Nanjing 210098, China*

c. *CSIRO Data 61, Private Bag 5, Wembley, WA 6913, Australia*

*Corresponding author:

Dr. Weiguang Wang

Email: wangweiguang006@126.com; wangweiguang006@gmail.com

The Supplementary Information included in our paper comprises:

1. Supplementary Table S1-S3.

3. Supplementary Figure S1-S9.

Supplementary Table S1 Before and after the first 11 rotate principal components of the variance contribution (%)

Component	Before rotation		After rotation	
	% of Variance	Cumulative %	% of Variance	Cumulative %
1	25.4	25.4	18.7	18.7
2	15.9	41.3	11.9	30.6
3	8.3	49.6	11.0	41.6
4	7.2	56.8	8.9	50.5
5	5.9	62.7	7.2	57.7
6	4.8	67.5	6.4	64.1
7	3.6	71.1	5.9	70.0
8	3.1	74.2	3.7	73.7
9	2.5	76.7	2.8	76.5
10	2.3	79.0	2.4	78.9
11	1.8	80.8	1.9	80.8

Supplementary Table S2 Contributions of climate factors to the trends in annual reference evapotranspiration (ET_0) in the eight climatic regions

Sub-region		Variables	Periods	
			1961-1991	*1992-2011*
EC	CALC	$C\_(T)$	-0.93	0.18
		$C\_(RH)$	-0.58	2.32
		$C\_(U)$	-0.66	-0.52
		$C\_(R_n)$	-1.28	-0.56
		$C\_(ET_0)$	-3.45	1.42
		$Trend(ET_0)$	-3.26	1.51
		δ	-0.18	-0.09
		$\rho(\delta)(\%)$	5.00	-6.00
		Dominating Factor	R_n	*RH*
			1961-1994	*1995-2011*
SE	CALC	$C\_(T)$	0.74	0.17
		$C\_(RH)$	0.16	3.11
		$C\_(U)$	-0.34	-0.13
		$C\_(R_n)$	-2.15	0.19
		$C\_(ET_0)$	-1.59	3.33
		$Trend(ET_0)$	-1.72	3.76
		δ	0.13	-0.42
		$\rho(\delta)(\%)$	-7.49	-11.23
		Dominating Factor	R_n	*RH*
			1961-1991	*1992-2011*
SW	CALC	$C\_(T)$	0.85	0.10
		$C\_(RH)$	0.22	1.64
		$C\_(U)$	-0.47	1.26
		$C\_(R_n)$	-1.90	0.24
		$C\_(ET_0)$	-1.30	3.24

Sub-region		Variables	Periods			
			1961-1972	*1973-1990*	*1991-1999*	*2000-2011*
NC	CALC	$C\_(T)$	-0.42	0.36	1.79	-0.59
		$C\_(RH)$	1.18	-0.43	2.95	0.41
		$C\_(U)$	2.53	-2.12	2.40	-0.38
		$C\_(R_n)$	0.85	-0.47	0.89	-1.60
		$C\_(ET_0)$	4.14	-2.66	8.02	-2.16
		$Trend(ET_0)$	4.42	-3.04	8.19	-2.54
		δ	-0.28	0.37	-0.17	0.38
		$\rho(\delta)(\%)$	-6.39	-12.27	-2.05	-14.78
		Dominating Factor	*U*	*U*	*RH*	R_n
				1961-1976	*1977-1994*	*1995-2011*
NW	CALC	$C\_(T)$		-0.97	0.51	0.49
		$C\_(RH)$		0.30	-1.86	1.58
		$C\_(U)$		1.95	-3.67	0.75
		$C\_(R_n)$		-0.65	-0.42	-0.19
		$C\_(ET_0)$		0.63	-5.44	2.63
		$Trend(ET_0)$		0.67	-5.97	3.09
		δ		-0.04	0.53	-0.46
		$\rho(\delta)(\%)$		-6.04	-8.80	-14.75
		Dominating Factor		*U*	*U*	*RH*
				1961-1991	*1992-2001*	*2002-2011*
NP	CALC	$C\_(T)$		0.29	1.83	-1.65
		$C\_(RH)$		0.10	2.19	0.37
		$C\_(U)$		-1.27	-0.96	-1.26
		$C\_(R_n)$		-0.53	0.95	-0.54
		$C\_(ET_0)$		-1.40	4.01	-3.08

Sub-region		Variables	Periods		
		Trend(ET_0)		-1.15	3.55
		δ		-0.15	-0.31
		$\rho(\delta)$(%)		13.18	-8.71
		Dominating Factor		R_n	*RH*
			1961-1972	*1973-1992*	*1993-2011*
		$C\_(T)$	0.45	0.14	0.74
		$C\_(RH)$	0.75	-0.31	2.02
	CALC	$C\_(U)$	3.46	-1.81	0.41
		$C\_(R_n)$	0.96	-0.31	-0.32
TP		$C\_(ET_0)$	5.63	-2.29	2.85
		Trend(ET_0)	5.16	-2.46	2.99
		δ	0.47	0.17	-0.14
		$\rho(\delta)$(%)	9.10	-7.05	-4.55
		Dominating Factor	*U*	*U*	*RH*

Sub-region		Variables	Periods			
		Trend(ET_0)		-1.38	4.37	-3.16
		δ		-0.02	-0.36	0.08
		$\rho(\delta)$(%)		1.70	-8.35	-2.62
		Dominating Factor		*U*	*RH*	*T*
			1961-1977	*1978-1992*	*1993-2002*	*2003-2011*
		$C\_(T)$	-0.44	0.59	-0.21	-1.13
		$C\_(RH)$	1.55	-0.92	3.45	-1.22
	CALC	$C\_(U)$	0.69	-1.87	-0.17	-2.32
		$C\_(R_n)$	0.15	-1.07	2.99	0.83
NE		$C\_(ET_0)$	1.95	-3.27	6.06	-3.84
		Trend(ET_0)	1.75	-3.57	6.59	-4.34
		δ	0.20	0.30	-0.53	0.50
		$\rho(\delta)$(%)	11.42	-8.43	-8.04	-11.54
		Dominating Factor	*RH*	*U RH*		*U*

Note : CALC: calculated trends by equation (10); the unit is given in mm yr^{-2}. 'δ' and '$\rho(\delta)$' mean error and relative error to observed trends, respectively. See Figure 1 for descriptions of climatic regions.

Supplementary Table S3 Calibration of the Angstrom coefficients for the 51 stations with radiation observations

WMO Number	Station Name	a_s	b_s	R^2	WMO Number	Station Name	a_s	b_s	R^2
50527	Hailaer	0.153	0.315	0.922	54662	Dalian	0.195	0.285	0.737
50873	Jiamusi	0.165	0.638	0.858	54823	Jinan	0.188	0.557	0.764
50953	Harbin	0.184	0.589	0.875	55591	Lhasa	0.217	0.315	0.543
51076	Altay	0.183	0.319	0.964	56029	Yushu	0.162	0.306	0.575
51431	Ghulja	0.204	0.295	0.904	56137	Changdu	0.175	0.451	0.568
51463	Urumqi	0.216	0.315	0.953	56651	Lijiang	0.156	0.305	0.594
51573	Turpan	0.189	0.218	0.948	56778	Kunming	0.135	0.398	0.686
51709	Kashgar	0.186	0.365	0.884	56959	Jinghong	0.167	0.257	0.523
51777	Ruoqiang	0.221	0.419	0.909	57083	Zhengzhou	0.189	0.493	0.867
51828	Hotan	0.205	0.395	0.887	57461	Yichang	0.200	0.504	0.830
52203	Hami	0.195	0.328	0.937	57494	Wuhan	0.174	0.631	0.774
52418	Dunhuang	0.228	0.486	0.845	57679	Changsha	0.165	0.659	0.886
52681	Minqin	0.227	0.501	0.864	57816	Guiyang	0.162	0.489	0.748
52818	Geermu	0.143	0.314	0.948	57957	Guilin	0.145	0.657	0.813
52866	Xining	0.206	0.621	0.726	57993	Ganzhou	0.205	0.528	0.888
53068	Erenhot	0.155	0.456	0.929	58208	Gushi	0.191	0.624	0.816
53487	Datong	0.158	0.547	0.89	58238	Nanjing	0.164	0.781	0.868
53614	Yinchuan	0.169	0.585	0.825	58321	Hefei	0.181	0.552	0.807
53772	Taiyuan	0.141	0.591	0.866	58457	Hangzhou	0.129	0.498	0.841
54135	Tongliao	0.208	0.317	0.762	58606	Nanchang	0.152	0.563	0.843
54161	Changchun	0.175	0.637	0.903	58847	Fuzhou	0.184	0.653	0.835
54292	Yanji	0.226	0.451	0.874	59287	Guangzhou	0.125	0.753	0.778
54324	Chaoyang	0.152	0.455	0.834	59316	Shantou	0.126	0.714	0.762
54342	Shenyang	0.148	0.517	0.875	59431	Nanning	0.136	0.587	0.815
54511	Beijing	0.152	0.775	0.913	59758	Haikou	0.174	0.471	0.735
54527	Tianjin	0.135	0.523	0.835					

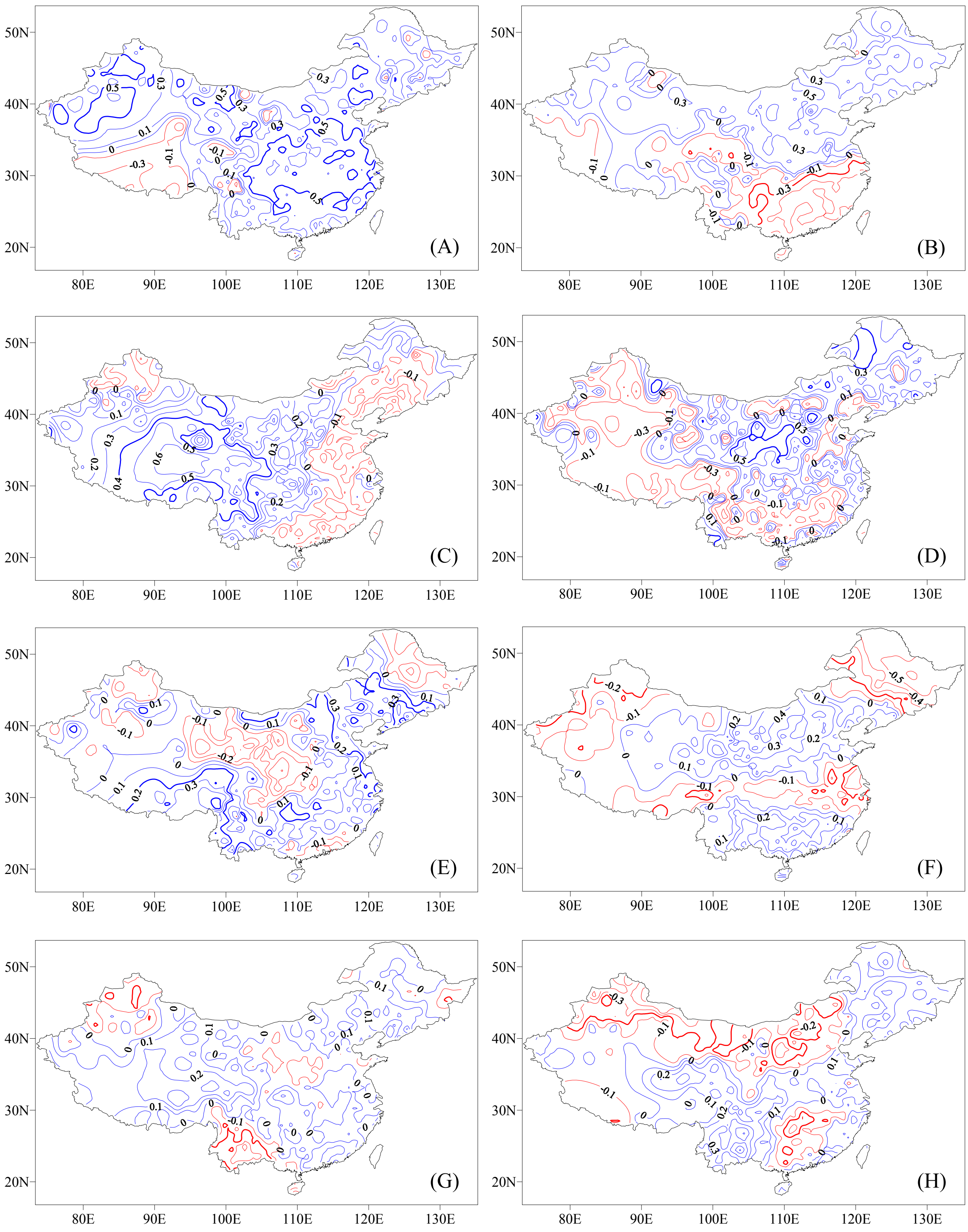

Supplementary Fig. S1. First 8 REOF modes of ET_0 with about 73.7% of total variation (A) first mode with about 18.7 of total variation (B) second mode with about 11.9 of total variation (C) third mode with about 11.0 of total variation (D) fourth mode with about 8.9 of total variation (E) fifth mode with about 7.2 of total variation (F) sixth mode with about 6.4 of total variation (G) seventh mode with about 5.9 of total variation (H) eighth mode with about 3.7 of total variation. This figure was created using the Golden Software Surfer 8.0 (http://www.goldensoftware.com/products/surfer/).

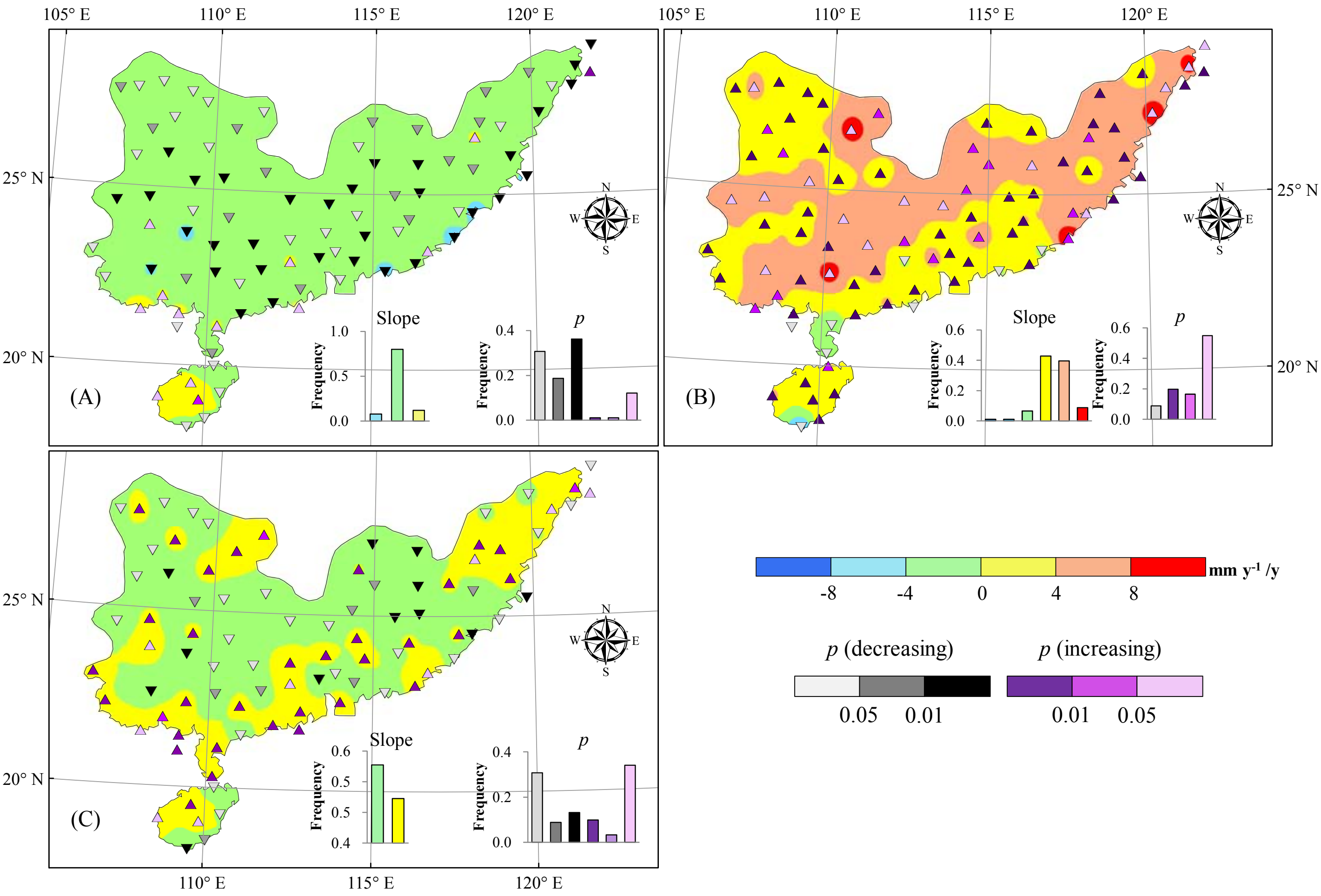

Supplementary Fig. S2. Spatial distributions of the change rate of reference evapotranspiration (ET_0) and significance levels of the change in the second sub-region (SE) during the three periods (A: 1961-1994; B: 1995-2011; and C: 1961-2011). *p*(decreasing) and *p*(increasing) are the *p* values of the decrease and increase in the annual reference evapotranspiration (ET_0), respectively, which are divided into three levels: $p < 0.01$, $0.01 < p < 0.05$, and $p > 0.05$. The insets show the frequency distributions of change trends of reference evapotranspiration (ET_0) (left) and different significance levels (right). This figure was created using the ArcGIS 9.3 (http://www.esri.com/software/arcgis/arcgis-for-desktop).

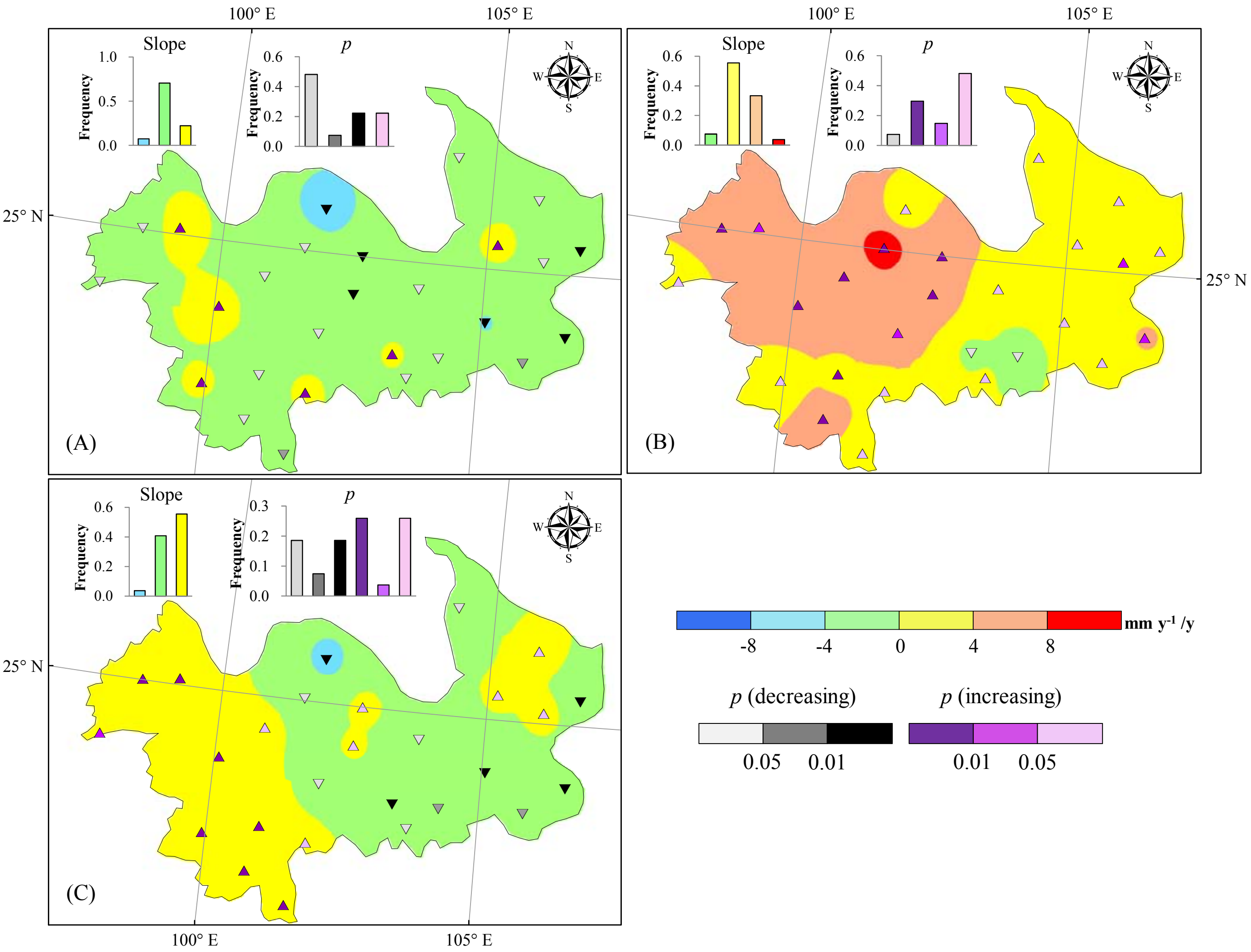

Supplementary Fig. S3. Spatial distributions of the change rate of reference evapotranspiration (ET_0) and significance levels of the change in the third sub-region (SW) during the three periods (A: 1961-1991; B: 1992-2011; and C: 1961-2011). *p*(decreasing) and *p*(increasing) are the *p* values of the decrease and increase in the annual reference evapotranspiration (ET_0), respectively, which are divided into three levels: $p < 0.01$, $0.01 < p < 0.05$, and $p > 0.05$. The insets show the frequency distributions of change trends of reference evapotranspiration (ET_0) (left) and different significance levels (right). This figure was created using the ArcGIS 9.3 (http://www.esri.com/software/arcgis/arcgis-for-desktop).

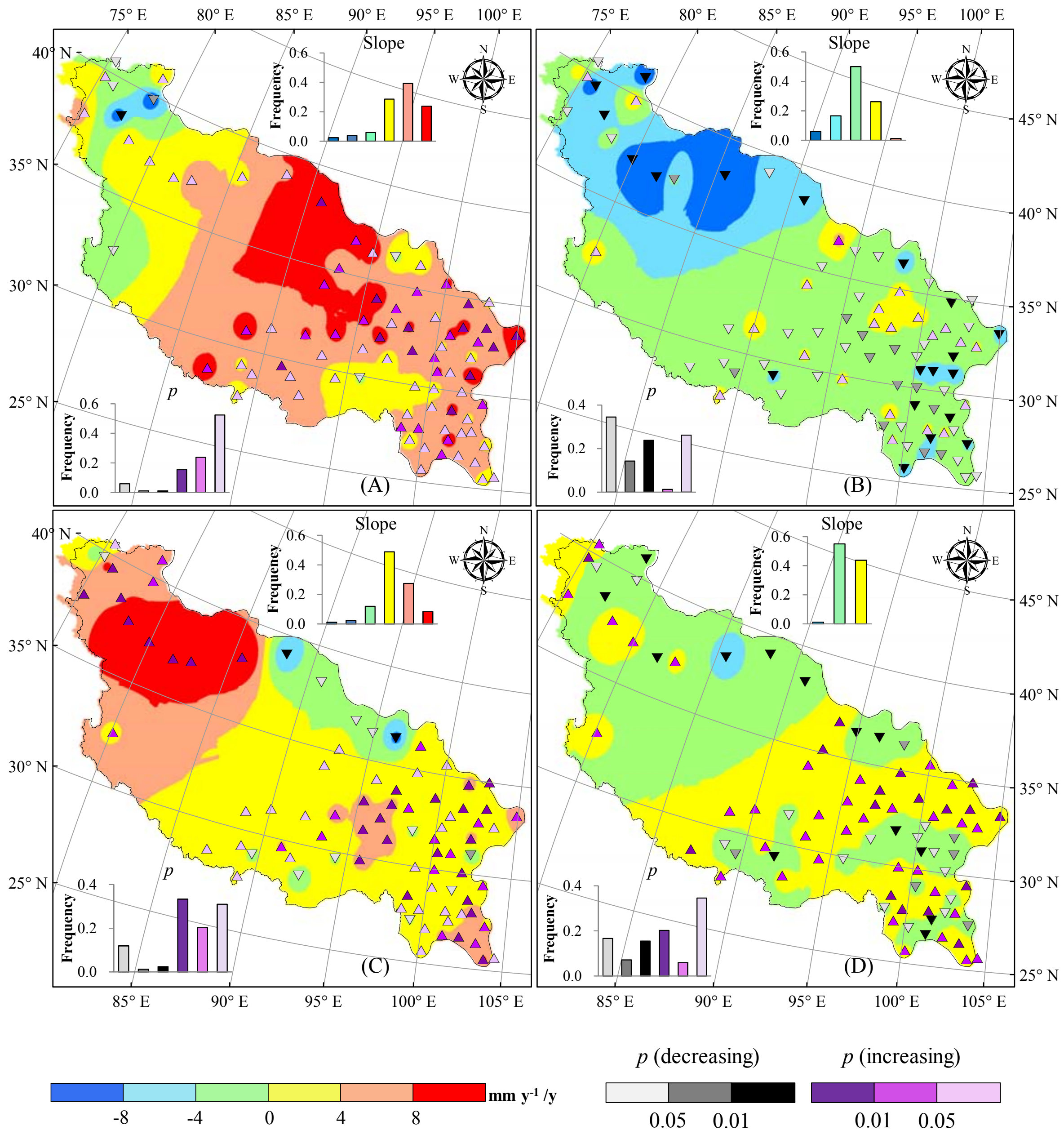

Supplementary Fig. S4. Spatial distributions of the change rate of reference evapotranspiration (ET_0) and significance levels of the change in the fourth sub-region (TP) during the four periods (A: 1961-1972; B: 1973-1992; C: 1993-2011 and D: 1961-2011). *p*(decreasing) and *p*(increasing) are the *p* values of the decrease and increase in the annual reference evapotranspiration (ET_0), respectively, which are divided into three levels: $p < 0.01$, $0.01 < p < 0.05$, and $p > 0.05$. The insets show the frequency distributions of change trends of reference evapotranspiration (ET_0) (left) and different significance levels (right). This figure was created using the ArcGIS 9.3 (http://www.esri.com/software/arcgis/arcgis-for-desktop).

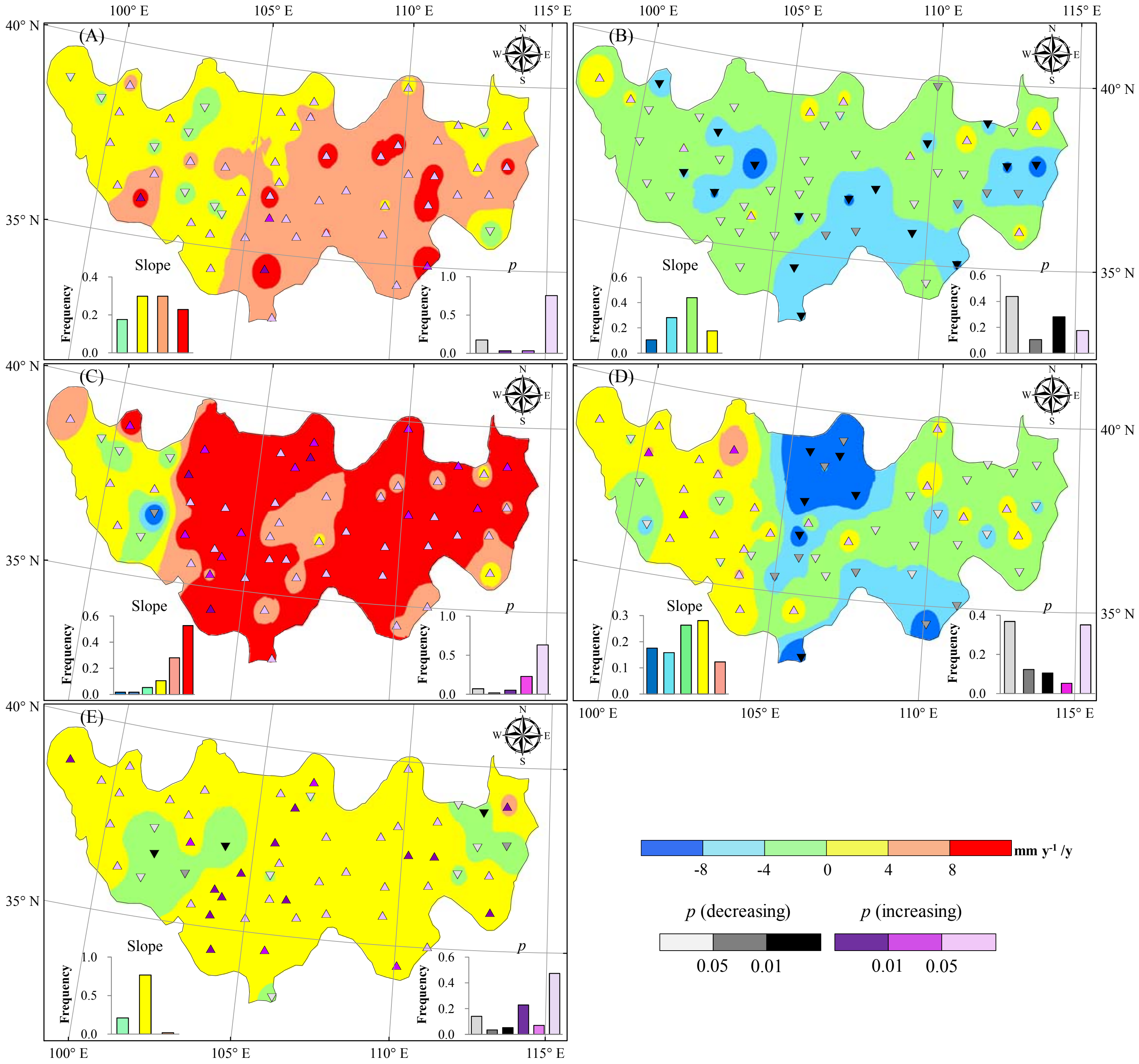

Supplementary Fig. S5. Spatial distributions of the change rate of reference evapotranspiration (ET_0) and significance levels of the change in the fifth sub-region (NC) during the five periods (A: 1961-1972; B: 1973-1990; C: 1991-1999; D: 2000-2011; and E: 1961-2011). *p*(decreasing) and *p*(increasing) are the *p* values of the decrease and increase in the annual reference evapotranspiration (ET_0), respectively, which are divided into three levels: $p < 0.01$, $0.01 < p < 0.05$, and $p > 0.05$. The insets show the frequency distributions of change trends of reference evapotranspiration (ET_0) (left) and different significance levels (right). This figure was created using the ArcGIS 9.3 (http://www.esri.com/software/arcgis/arcgis-for-desktop).

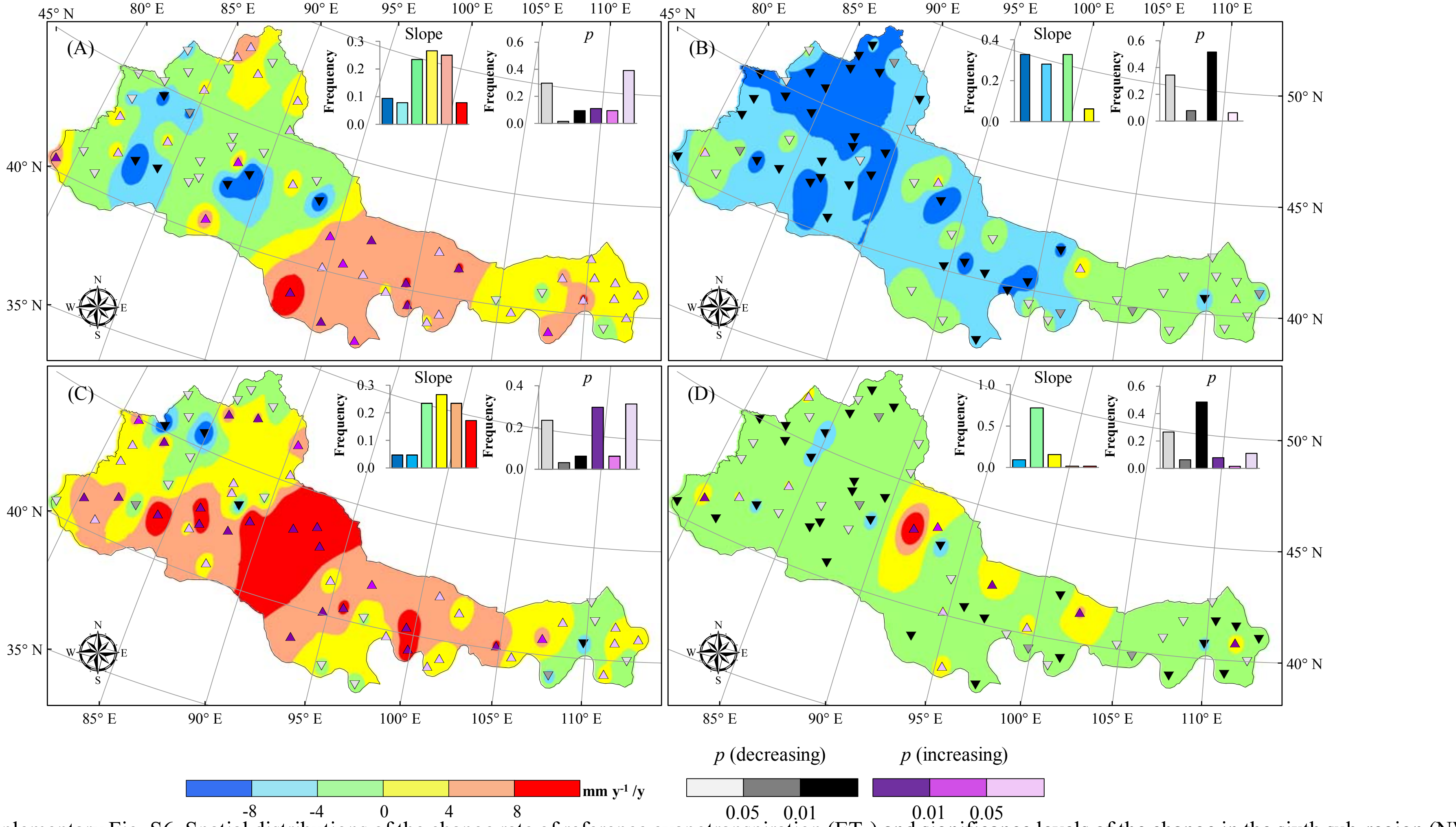

Supplementary Fig. S6. Spatial distributions of the change rate of reference evapotranspiration (ET_0) and significance levels of the change in the sixth sub-region (NW) during the four periods (A: 1961-1976; B: 1977-1994; C: 1995-2011; and D: 1961-2011). *p*(decreasing) and *p*(increasing) are the *p* values of the decrease and increase in the annual reference evapotranspiration (ET_0), respectively, which are divided into three levels: $p < 0.01$, $0.01 < p < 0.05$, and $p > 0.05$. The insets show the frequency distributions of change trends of reference evapotranspiration (ET_0) (left) and different significance levels (right). This figure was created using the ArcGIS 9.3 (http://www.esri.com/software/arcgis/arcgis-for-desktop).

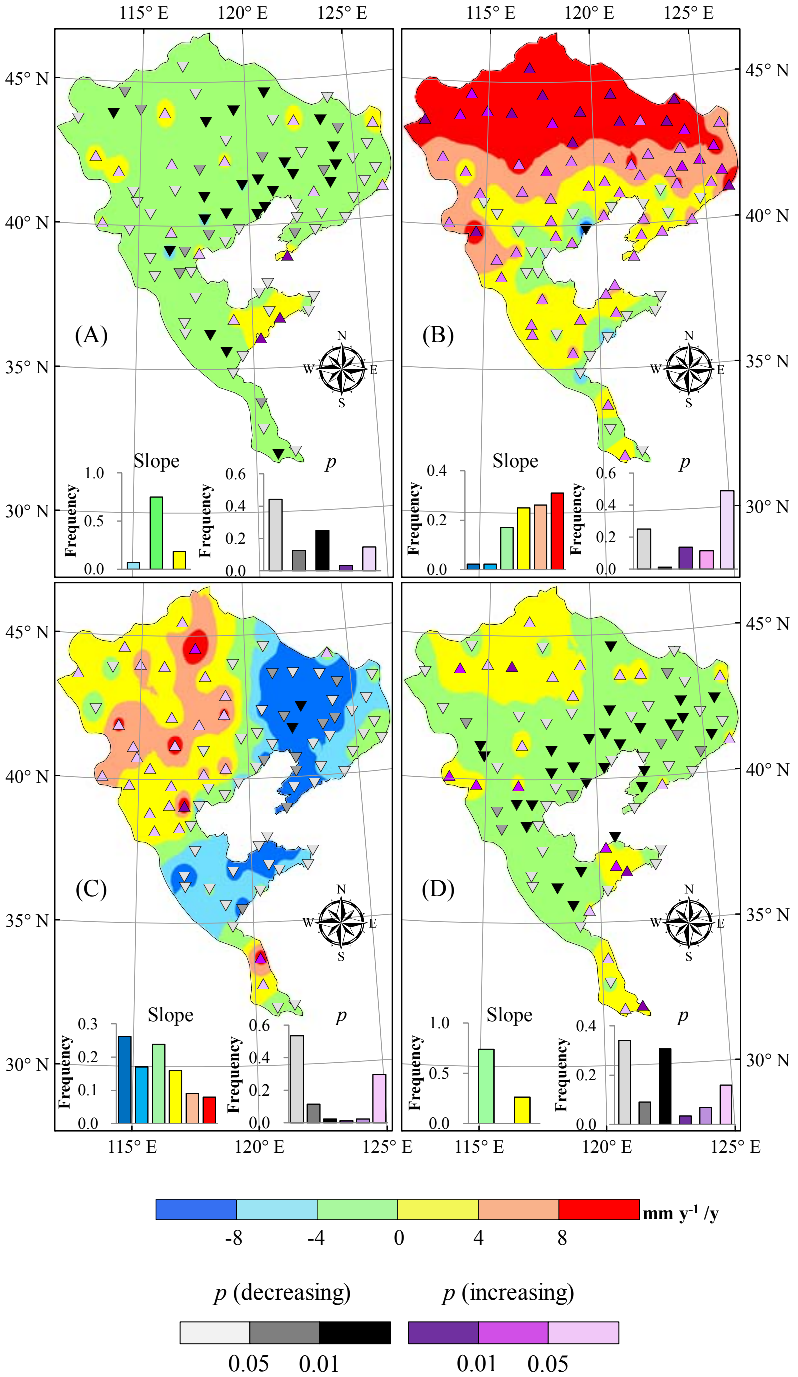

Supplementary Fig. S7. Spatial distributions of the change rate of reference evapotranspiration (ET_0) and significance levels of the change in the seventh sub-region (NP) during the four periods (A: 1961-1991; B: 1992-2001; C: 2002-2011; and D: 1961-2011). *p*(decreasing) and *p*(increasing) are the *p* values of the decrease and increase in the annual reference evapotranspiration (ET_0), respectively, which are divided into three levels: $p < 0.01$, $0.01 < p < 0.05$, and $p > 0.05$. The insets show the frequency distributions of change trends of reference evapotranspiration (ET_0) (left) and different significance levels (right). This figure was created using the ArcGIS 9.3 (http://www.esri.com/software/arcgis/arcgis-for-desktop).

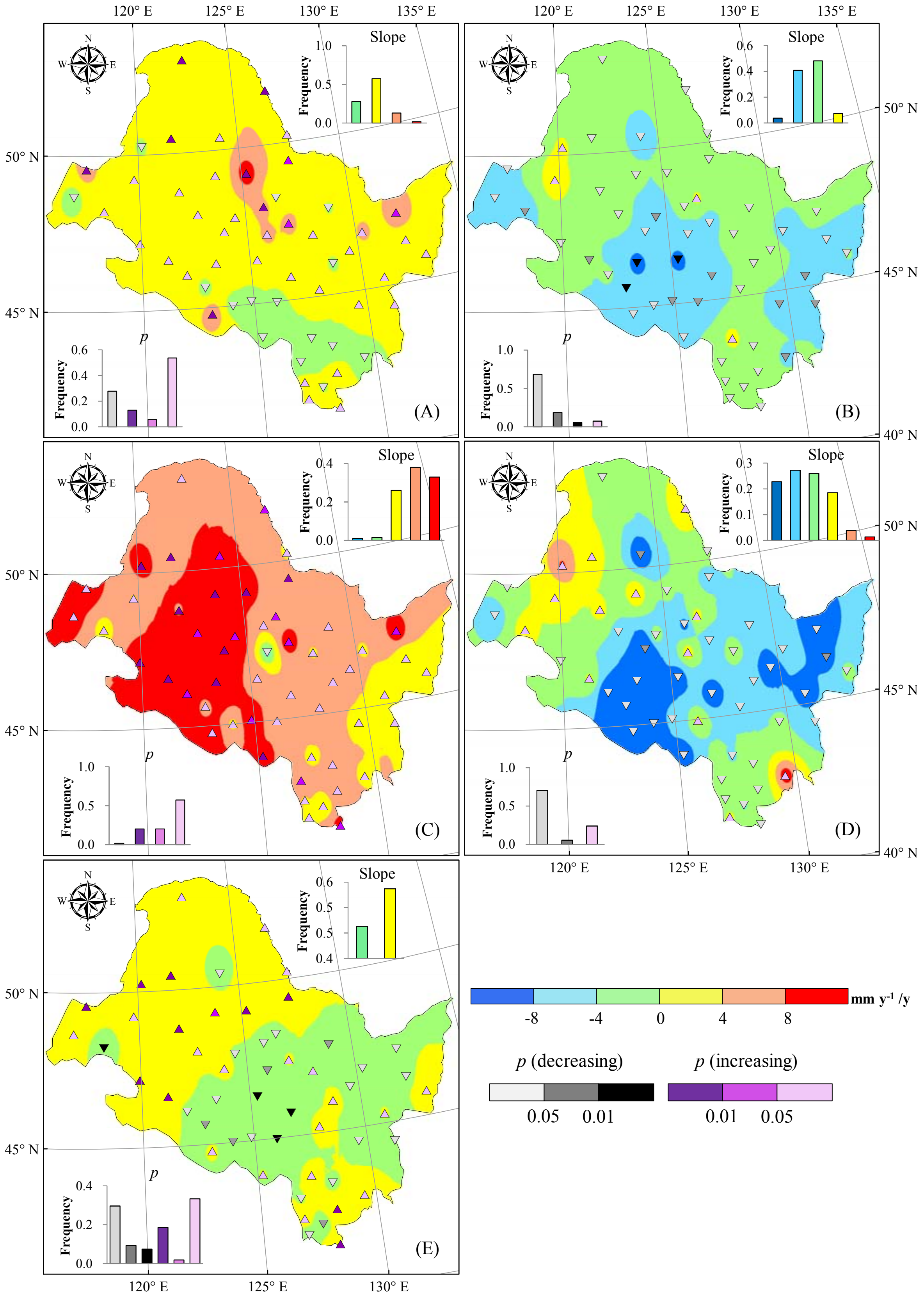

Supplementary Fig. S8. Spatial distributions of the change rate of reference evapotranspiration (ET_0) and significance levels of the change in the eighth sub-region (NE) during the five periods (A: 1961-1977; B: 1978-1992; C: 1993-2002; D: 2003-2011; and E: 1961-2011). *p*(decreasing) and *p*(increasing) are the *p* values of the decrease and increase in the annual reference evapotranspiration (ET_0), respectively, which are divided into three levels: $p < 0.01$, $0.01 < p < 0.05$, and $p > 0.05$. The insets show the frequency distributions of change trends of reference evapotranspiration (ET_0) (left) and different significance levels (right). This figure was created using the ArcGIS 9.3 (http://www.esri.com/software/arcgis/arcgis-for-desktop).

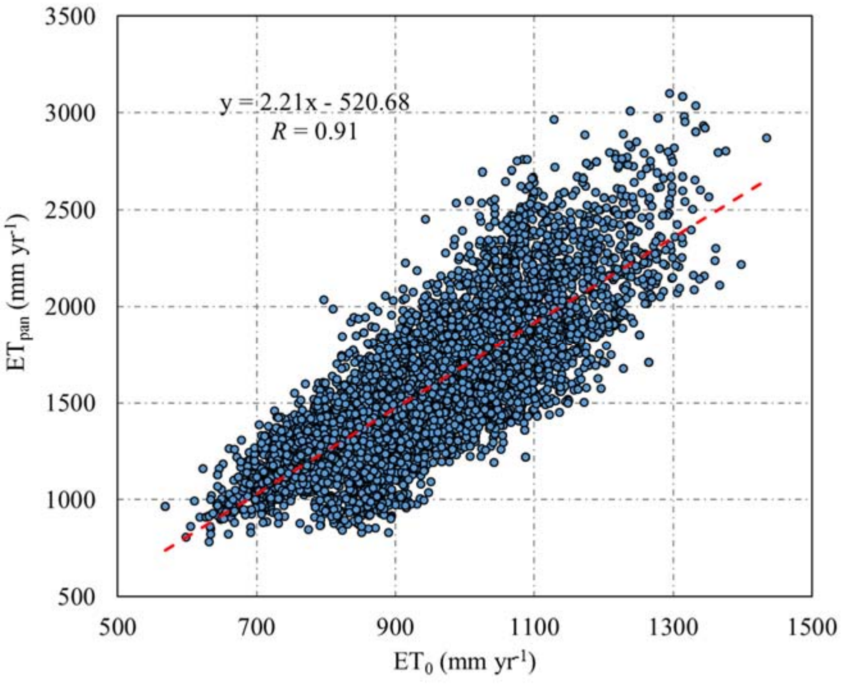

Figure S9 Relationship between pan evaporation (ET_{pan}) and reference evapotranspiration (ET_0).
